# Seductive or deceiving? A focus group study on perspectives and expectations of consumers on meat alternative labeling practices

**DOI:** 10.3389/fpubh.2026.1938629

**Published:** 2026-09-22

**Authors:** Linsay Ketelings, Roos Caanen, Remco C. Havermans, Stef P. J. Kremers, Alie de Boer

**Affiliations:** 1 Food Claims Research Venlo, System Earth Science, Faculty of Science and Engineering, Maastricht University Campus Venlo, Venlo, Netherlands; 2 Laboratory of Behavioural Gastronomy, System Earth Science, Faculty of Science and Engineering, Maastricht University Campus Venlo, Venlo, Netherlands; 3 School for Nutrition and Translational Research in Metabolism (NUTRIM), Department of Health Promotion, Maastricht University, Maastricht, Netherlands

**Keywords:** consumer behavior, European food law, food labeling, food systems, plant-based meat alternatives, qualitative research, sustainability

## Abstract

**Introduction:**

As the global food system seeks sustainable solutions to address the challenges of climate change and a growing population, meat alternatives have emerged as a promising option for reducing the environmental impact of meat production. Over the past decade, their promotion has increased, while debates persist regarding the use of meat-related terminology on these products and its potential to confuse or mislead consumers. This study investigates Dutch consumers’ perceptions of meat alternatives, with particular emphasis on elements perceived as confusing or misleading.

**Methods:**

A focus group study was conducted among Dutch consumers to explore their perceptions and experiences with meat alternatives and current labeling practices using a reflexive thematic analysis approach. A total of five focus groups were conducted, with participant compositions differing according to their level of meat consumption.

**Results:**

Product naming, positioning, and credence attributes were identified as key factors shaping consumer expectations. Although participants did not report unintentionally purchasing meat alternatives, they expressed concerns about potentially feeling misled by current labeling practices. At the same time, participants recognized that meat-like terminology can enhance familiarity and provide useful reference points regarding sensory properties, preparation, and usage.

**Discussion and conclusion:**

The findings highlight a distinction between legal and societal forms of misleading, emphasizing the need for clear and transparent communication across the food chain. Regulatory frameworks and policy efforts may play a critical role in standardizing labeling practices to ensure that information on meat alternatives is both informative and trustworthy. Balancing consumer protection with effective communication is essential for fostering consumer trust and supporting the wider adoption of sustainable meat alternatives in line with global sustainability goals.

## 1 Introduction

The consumption of plant-based meat alternatives has increased over the past decade (1). Not only consumers with a vegan or vegetarian diet, but also flexitarian and omnivorous individuals consume more meat alternatives with the aim of reducing their meat consumption (2, 3). Meat alternatives, also known as ‘meat substitutes’, ‘meat analogues’ or ‘meat replacements’, are plant-based food products that aim to imitate the sensory and nutritional aspects of meat and are explicitly intended to replace meat as a meal component (4).

The growing popularity of meat and dairy alternatives has intensified public and political debate on the regulation of alternative protein labeling across different jurisdictions (5). One thoroughly discussed issue is whether plant-based products should be allowed to use terms traditionally associated with animal-derived foods, such as burger, sausage, steak, or milk. While proponents argue that these familiar terms help consumers understand how products are prepared and consumed, opponents contend that they may mislead consumers and undermine the value of animal-derived products (6).

Within the European Union (EU), dairy and meat alternatives are governed under different regulatory regimes. For dairy alternatives, Regulation (EU) No 1308/2013 (the Common Organisation of the Market (COM) Regulation) reserves designations such as milk, cheese, butter, and yoghurt exclusively for products of animal origin (7). This interpretation was confirmed by the Court of Justice of the European Union in the 2017 *TofuTown case*, which held that plant-based products may not use protected dairy designations, even when accompanied by clarifying terms such as “soy” or “plant-based” (8). By contrast, the regulation of meat-related terminology has remained more contested. In 2019, the European Parliament’s Committee on Agriculture and Rural Development (AGRI) proposed restricting the use of terms such as burger and sausage for plant-based products, but the proposal was rejected by the European Parliament in 2020 (9). Subsequently, France introduced a decree prohibiting the use of certain meat-related terms for plant-based foods. However, in 2024, the Court of Justice of the European Union ruled that, in the absence of harmonized EU legislation, Member States could not unilaterally prohibit the use of such terminology (10). Following renewed legislative efforts at the EU level, a voting in 2025 passed in the European Parliament to reserve certain meat-related terms for products of animal origin (particularly names of animal species and specific cuts of meat, such as beef, chicken, steak, and bacon). At the same time, generic culinary terms such as burger, sausage, and nuggets remain permissible for plant-based products, provided their vegetarian or vegan nature is clearly indicated (11, 12). The provisional agreement still requires approval from the Council of the EU before the new rules can enter into force. Despite continued debate, these developments reflect the EU’s attempt to strike a balance between consumer information, fair competition, the interests of livestock producers, and the use of familiar product descriptors that support consumer understanding.

The distinction between confusion and misleading is central to the debate on the labeling of meat alternatives, as EU food law requires that food information does not mislead consumers (13). However, the two terms are often used interchangeably in legislation and policy discussions, despite referring to different concepts. EU food law does not provide a clear definition distinguishing them. In this context, confusion can be understood as a cognitive response in which consumers are uncertain or unclear about the information presented to them, whereas misleading information refers to information that is presented or used in a way that may lead consumers to develop an inaccurate or false perception of a product (14). Importantly, information does not necessarily have to be factually false to be misleading. Even technically accurate information can be presented in a way that creates a false perception or influences consumers’ judgements about a product (14). Confusion may therefore be one mechanism through which consumers can ultimately be misled, although confusion does not necessarily imply that consumers have been misled.

Little previous research has examined how consumers interpret meat-related terminology on plant-based meat alternatives, with mixed findings. While some studies suggest that consumers generally understand such terminology as referring to the use or preparation of the product, other research indicates that meat-related terminology can cause some degree of confusion or ambiguity (15–19). Recent evidence further suggests that meat-like terminology may affect consumers’ ability to correctly identify the nature of a product. Specifically, the use of meat-like names on meat alternative labels resulted in more errors when consumers were asked to classify products as plant-based or animal-based (16). These findings suggest that consumer responses to meat-related terminology are nuanced and may depend on the broader labeling and product context. They also highlight the importance of distinguishing between confusion and misleading: evidence that consumers experience difficulty or uncertainty in interpreting a label does not, in itself, demonstrate that the label has misled consumers. Whether meat-related terminology on plant-based products actually leads to confusion or misleading perceptions therefore remains an empirical question. Although concerns about consumer misunderstanding have played a central role in regulatory and political debates on meat alternative labeling, relatively little is known about how consumers themselves interpret these terms in practice and what aspects of labeling contribute to perceptions of confusion or misleadingness. Therefore, the aim of this study was to investigate which aspects of meat alternative labeling are perceived as confusing and/or misleading by Dutch consumers. This was examined using focus groups to obtain in-depth insights into consumers’ experiences and perceptions of the labeling of meat alternatives.

## 2 Methods

### 2.1 Participants

A total of 25 participants were recruited through social media posts, flyers, and convenience sampling (20). Participants did not receive a financial incentive; however, refreshments were provided free of charge, and participants were eligible for reimbursement of travel expenses. To obtain diverse in-depth insights, three different focus groups compositions were made based on participants’ average frequency of meat consumption at the main meal as indicated upon accepting the invitation to participate in the study: (1) non-regular meat consumers, who consumed meat at the main meal no more than twice a week; (2) regular meat consumers, who consumed meat at the main meal more than twice a week; and (3) a mixed group consisting of both regular and non-regular meat consumers. This composition allowed for a more in-depth exploration of perceptions among participants with similar meat consumption patterns in groups 1 and 2, while the mixed composition of group 3 was intended to elicit discussion between participants with different consumption patterns. This resulted in a total of five focus groups consisting of four to six participants. An overview of the focus groups’ composition and participants’ demographics can be found in Table 1. These demographics were included as they are found to influence meat (alternative) perceptions and consumption, including age, diet, gender, religion, and education level (16, 21).

**Table 1:** Focus group compositions and participant demographics.

Participant number	Age	Gender	Highest obtained educational degree	Degree of meat consumption
Focus group 1–Non-regular meat consumption^1^				
P126	55	Female	Bachelor	None
P147	26	Female	Master	None
P163	26	Female	Master	1–2 times a week
P179	34	Female	PhD	None
P181	42	Female	Bachelor	None
Focus group 2–Regular^2^ and non-regular meat consumption				
P195	20	Female	Post-secondary vocational education	None
P255	22	Female	Bachelor	1–2 times a week
P265	24	Male	Bachelor	Every day
P280	53	Female	Post-secondary vocational education	1–2 times a week
P292	56	Male	Bachelor	Every day
Focus group 3–Regular meat consumption				
P310	26	Male	Bachelor	Every day
P327	66	Female	Secondary education	Every day
P357	55	Female	Secondary education	3–6 times a week
P386	65	Male	Post-secondary vocational education	Every day
Focus group 4–Regular and non-regular meat consumption				
P391	23	Female	Bachelor	1–2 times a week
P406	19	Female	Post-secondary vocational education	Every day
P437	52	Male	Master	1–2 times a week
P444	52	Female	Bachelor	3–6 times a week
P483	24	Female	Bachelor	Every day
Focus group 5–Non-regular meat consumption				
P498	22	Male	Bachelor	None
P511	24	Female	Bachelor	None
P523	22	Female	Bachelor	None
P569	23	Male	Bachelor	None
P570	22	Female	Bachelor	None
P582	24	Female	Bachelor	None

^1^ Non-regular meat consumption = Meat consumption frequency during main meal: max. 2 times a week. ^2^Regular meat consumption = Meat consumption frequency during main meal: >2 times a week.

### 2.2 Data collection

The in-person focus group discussions lasted between 90 and 120 min and were held in different cities in the Netherlands in 2022 and 2023. Data was collected in the form of audio recordings using two Olympus digital voice recorders (WS-853), accompanied by notes and observations by the moderator and assistant present during the focus groups.

Prior to the start of the in-person focus group discussion, participants received a verbal introduction to the study. Subsequently, participants had to sign the written informed consent form, after which they were asked to fill in a short demographic survey. For the purposes of this study, participants were made aware that a meat alternative was defined as “any pre-packaged meat alternative in the designated meat alternative section of supermarkets”.

Each focus group was executed in three phases based on a semi-structured discussion guide. In phase one, a set of open-ended questions was used to stimulate a discussion on participants’ general knowledge and experience with meat alternatives. The second phase focused on the presentation and labeling of meat alternatives, and potential confusion and misleading. The focus groups concluded with a discussion of two commercially available meat alternatives; one resembling meat and one not resembling meat. These products were introduced with the aim to engage participants and possibly elicit more detailed or specific perceptions of the topic, in addition to those already obtained in the second phase (22). The full discussion guide is available in the Supplementary materials.

### 2.3 Data analysis

The audio recordings were first transcribed *verbatim* by author RC, during which participant names were replaced with alternative identifying terms using randomized three-digit numbers. Next, a Reflexive Thematic Analysis (RTA) was conducted based on the six-step process as specified by Braun and Clarke (23). This approach was deemed appropriate because the study aimed to explore and interpret consumers’ perceptions and experiences regarding the studied topic, acknowledging that these are shaped by individual and contextual factors. Reflexive thematic analysis therefore provided a suitable methodological approach to generate an in-depth understanding of participants’ perspectives (24). A thematic analysis (TA) provides a “systematic approach for identifying, analyzing, and reporting patterns – themes – across a dataset through processes of coding” (22). In this study, the flexibility of TA allowed for an in-depth exploration of consumers’ perceptions and experiences using a combination of inductive and deductive methods (23, 25). In an exclusively inductive analysis approach, an analysis is generated ‘bottom-up’, with codes and themes grounded in the acquired data (26). On the contrary, in an exclusively deductive analysis approach, an analysis is generated ‘top-down’ to explore theoretical ideas, with codes and themes created prior to the analysis. The use of TA therefore enabled an analysis that aimed to identify patterns of meaning in the collected data, while simultaneously exploring findings from existing literature on the studied topic (22).

A characteristic differentiating RTA from other TA approaches is the central role of researcher reflexivity, which involves acknowledging the influence of the researchers in data collection, analysis and interpretation processes and making this transparent (23). As part of this study’s analysis, the researchers RC and LK reflected upon their personal background and stance on the studied topic throughout the study, as well as on the potential impact of these factors throughout the study. A researcher reflexivity statement can be found in the Supplementary materials. ATLAS.ti version 9.1.7.0 was used for the thematic analysis. The authors LK and RC independently read the transcripts line-by-line, and any relevant words, clusters, sentences, or sections were identified and coded using the constant comparative method. This method required continuous comparison between new and previously coded data abstracts to identify similarities and differences until the data was exhausted. The codes were named after terminology used by participants during the focus groups, also known as in-vivo coding. Additionally, prior to the focus groups, a set of deductive codes was developed based on findings from the existing literature identified in our previously conducted scoping review (4). These deductive codes were applied after the initial inductive coding round to explore concepts and findings reported in previous studies and were subsequently refined based on the inductive codes emerging from the data. Afterwards, all generated codes were further defined by distinguishing and clustering codes and subcodes into a codebook by authors LK and RC (23).

In the consecutive stage, relevant patterns were evaluated within the data and overarching themes were created based on their perceived importance. The codes in the codebook were assigned to the different themes accordingly, after which these themes were evaluated, interpreted, and visually structured in a ‘thematic map’ based on their interconnectedness and relevance regarding the studied topic (22, 23).

The study is reported using the Consolidated criteria for Reporting Qualitative research (COREQ) (27).

### 2.4 Ethical considerations

There were no risks or harms associated with this study. Prior to conducting the research, any procedures that were used in this study were approved by the Ethical Review Committee Inner city (ERCIC) from Maastricht University (ERCIC_327_16_02_2022).

## 3 Results

Three main themes were generated from the data when analyzing the answers to the question whether (labels of) meat alternatives are confusing and/or misleading. These include: 1. “Possible misleading elements”, 2. “Expectation management” and 3. “Who is responsible?”. Additionally, subthemes were created to obtain more depth into these three main themes. Main themes and subthemes were structured and visualized in a thematic table (Table 2). In the following sections, these themes are further clarified. Differences related to meat consumption frequency are reported where they emerged as relevant within the identified themes.

**Table 2:** Themes and subthemes.

Themes	Subthemes
Possible misleading elements	1.1 Product name
	1.2 Product positioning
	1.3 Credence attributes
Expectation management	2.1 Expectations
	2.2 Unclarities
Who is responsible?	3.1 Authorities
	3.2 FBOs and retailers
	3.3 Consumers

### 3.1 Theme 1: possible misleading elements

In all of the focus groups, the possibility for meat alternatives to be misleading arose. Several elements were observed that could be considered confusing or even misleading according to the participants. Within this theme, three subthemes were identified:

#### 3.1.1 Product name

Some participants observed a dichotomy within the meat alternatives product category currently on the market. On one hand, there are meat alternatives that strive to resemble meat and on the other hand products that do not try to resemble meat at all. One participant described this as follows:

*“Really a dichotomy, on one hand you have the products that want to replicate the meat (…) so mince and chicken, for example. And then you also have (…) a meat alternative that does not pretend to resemble meat, for example a veggie patty”* (P163).

Participant agreed that meat alternatives that do not try to resemble meat are not misleading and described that the product name clearly indicates that the product is not meat. Although a product name without any referral to meat was sometimes considered as an unappealing or unattractive product. Opinions on meat alternatives names that try to resemble meat varied highly. Some participants consider the use of meat-like terminology in the product name to be misleading:

*“The name should not be misleading; I agree with that. You need to know you are buying a vegetarian or vegan product. It should not be that you realize at home you bought a vegan alternative instead of meat and think the label misled me.”* (P265).

Other participants were not bothered much by these names and only saw how it could work positively:

*“(…) it* [the product name] *could make it easier to relate it to a specific product. So if I would want to make a curry with vegetarian chicken pieces, I am going to look for something called like that. But you need to see clearly that the product is not made from chicken.”* (P147).

Next, to the product name, the explicitness of the meat-free nature on the label of meat alternatives is a factor that influences the distinguishability between meat and meat alternatives. Whereas some participants perceived this as always being clearly indicated, others mentioned the opposite. Many participants agreed that the clarity of the meat-free nature of meat alternatives on their packaging or labeling is an important condition to avoid misleading.

*“With the names I do not have an issue (…) it is in a different section, and the packaging looks different (…) also the spelling is not the same (…) so I think that that is not per se misleading”* (P163).

#### 3.1.2 Product positioning in a retail environment

The participants indicated that they never bought a meat alternative with the assumption it was meat. The absence of any unintended meat alternative purchases seemed to be related to the position of meat alternatives in the supermarket. This was often explained by the separation of meat and meat alternatives in the supermarket:

*“As long as you have clearly different shelves (…) with ‘vegan’ and ‘chicken’ clearly indicated above them, that confusion won’t actually happen”* (P483).

In the hypothetical scenario that meat and meat alternative products are positioned next to each other, participants could imagine that consumers would purchase an alternative instead of meat by mistake:

*“…one big shelf where you cannot make the distinction anymore. You could possibly see something like I wanted to buy mince, but I came home with vegetarian mince.”* (P181).

Participants mentioned two exceptions in the supermarket in which meat and meat alternatives are placed in the same section: the freezer compartment and the spreads section. Some participants perceived the freezer section as a potential source of confusion, as the packaging of meat and meat alternatives was perceived to be very similar, making them feel that they could more likely select a meat alternative instead of meat by accident, although this had not happened to them. It could therefore be that the positioning of the meat alternative products in the supermarket influences the understanding and interpretation of said products.

#### 3.1.3 Credence attributes

Food products have attributes that cannot be judged independently by consumers, such as the time of sale through search (e.g., price or appearance) or experience (e.g., taste or texture). These attributes are also known as credence attributes (28). Other examples are environmental sustainability and the overall healthiness of a product. To exemplify, a consumer cannot assess how sustainable a meat alternative is without additional information from the Food Business Operators (FBOs) and/or retailer. Even if this information is provided, consumers have to assume this information is truthful.

In the focus groups, several credence attributes were observed that might or might not be misleading. One of these is how sustainable the meat alternative product really is compared to meat or other alternatives. Most participants initially assumed that meat alternative products are more sustainable than meat. But after a group discussion and giving it some thought, participants realized it is unclear and difficult to assess how sustainable a meat alternative is as information about this is missing on the label.

“*While we actually don’t know at all how sustainable the product really is, how much energy does it take to produce and what ingredients are actually in it? How sustainable are all these ingredients?”* (P181).

Another credence attribute that participants considered unclear was how healthy meat alternatives are. Participants mentioned certain nutrition claims lead to health expectations, as well as overall packaging or labeling looking healthy. Some participants considered it misleading when meat alternatives are promoted as being healthy, both directly and indirectly through suggestive marketing, as they indicated that this was not necessarily the case.

*“I think they are misleading yes, with the claims on the packaging they pretend a certain health aspect and a responsible diet”* (P327).

Related to healthiness, participants are puzzled by ingredients of meat alternatives, including their meaning and origin. This information is present on the label with the ingredient declaration, but participants do not always understand what they are. This uncertainty with regards to what a product is made of appears to be connected to the perceived processed nature of these products. Participants frequently described meat alternatives as being chemical, processed, or having many ingredients. However, this was counteracted by an argument that this is the case with many products found in the supermarket:

*“It is maybe misleading that you don’t know what’s in it or how it’s made.”* (P498).

*“I don’t think that’s a strong point because that is [the case] with a lot of products in the supermarket”* (P575 in response to P498).

### 3.2 Theme 2: expectation management

Information provided on the label (along with branding and marketing) can elicit expectations on different aspects of meat alternatives. This second theme discusses these expectations and whether these expectations are legitimate or whether unclarities remain.

#### 3.2.1 Expectations of meat alternatives

Different expectations can be set by a meat alternative label, for example, related to the sensory experience, nutritional value, and sustainability of the product. The use of green colors on the meat alternative packaging was associated with better for the planet and healthier compared to meat. Also, using meat-like terminology on meat alternatives like ‘beef’, ‘meatballs’, and ‘sausage’ can create expectations on the sensory experience.

*“I indeed think you start comparing more. If you call something [vegetarian] chicken pieces you think it should taste like chicken (…) If it is a veggie burger, I prepare for a veggie burger and not a hamburger”* (P406).

Opinions differed between participants about the usage of meat-like terminology on the label compared to non-established terminology. One demographic factor that appears to influence these opinions was the meat consumption frequency. This corresponds to consumers who are used to eating meat, desiring a meat-like experience when replacing meat, and consumers who seldomly or never eat meat, not desiring a meat-like experience (anymore). Consumers who seldomly consume meat appear unbothered by the use of meat-like terminology or even find it amusing.

*“I think it is funny how they play with the names [of meat alternatives]*. *Then I think: oh, that is clever!”* (P126).

However, most consumers regularly eating meat expressed perceiving this meat-like terminology of meat alternatives as being unattractive or unappealing. Furthermore, these participants perceived meat-like terminology as being tricky, complicated, or sometimes believed it to be misleading. Some expressed a lack of understanding of the reasoning behind such terminology.

*“I think it is a pity that it is approved that you can replace anything with a meat name (…) it still can be called bacon strips or chicken. Only it is vegetarian, it does not contain chicken. Then you are going to fool the people”* (P386).

Nevertheless, the use of meat-like terminology on the label elicits expectations about the meat-like experience, and if these are not met, it was perceived as a disadvantage:

*“I think it is very inconsistent, if you see oh, they indeed tried to recreate a piece of animal meat and then it is a bit disappointing. (…) If they try to mimic it and it did not work out, I don’t like it at all.”* (P195).

#### 3.2.2 Unclarities

Participants perceived different uncertainties and unclarities about meat alternatives. It became apparent that although participants had certain expectations about meat alternatives, they were often unsure on the accuracy of their expectations. When discussing their perceptions of meat alternatives, one participant expressed this uncertainty by referring to meat alternatives as being an enigma. This was also reflected in the fact that participants called for more transparency and information about meat alternatives in general, as well as more specific information on the label, for example in the form of certification or quality marks.

*“Indeed, maybe a quality mark or something where you can find where the ingredients or something are coming from”* (P310).

To a certain extent, the unclarities participant perceived are linked to the credence attributes as discussed in section 3.1.3. For example, participants are not certain how sustainable or healthy the meat alternatives truly are compared to meat. In the focus groups participants therefore also talked about transparency to remove these unclarities:

*“I think they* [FBOs] *should be more transparent”* (P444) *“Different packaging yeah, but if the packaging helps me with more detailed information about how healthy it is, that makes it easier for me because that is not my profession.”* (P437, in response to P444) *“I think about it the same as P437, it is just the unawareness.”* (P483, in response to P437).

### 3.3 Theme 3: who is responsible?

The third theme addresses which entities are involved in the discussion surrounding the question who is responsible for meat alternatives to not be misleading. This theme was not explicitly addressed in the questions asked in the focus groups but did become apparent during the discussions taking place. In the focus groups discussion, three groups of responsibility-holders were identified that are involved in not being misled by meat alternative labeling, namely: (i) authorities (EU + Member States), (ii) FBOs and retailers, and (iii) consumers.

#### 3.3.1 Authorities

Several participants mentioned the role the EU and national governments have in meat alternative consumption and marketing. There are several governmental strategies initiated to get consumers to eat less meat to reach climate goals. One participant noted that currently there is some discrepancy:

*“There is a lot of advertisement to stimulate meat consumption, also from the European Union, or [funded] with money from the EU. (…) While the European Union also has plans to be more sustainable. I do not understand this, there is a possibility to promote meat alternatives more.”* (P523) *“Similar that they cannot market cigarettes anymore (…) But I do not think it is weird if that is the case with meat as well”* (P575).

Accordingly, there is some responsibility expected from the EU to get people to eat less meat by for example imposing restrictions on the marketing of meat.

#### 3.3.2 FBOs and retailers

Participants believed that FBOs bear the greatest responsibility to not mislead consumers with their products and provide the correct information about the meat alternative. Despite these considerations, one participant mentioned that companies should be more transparent and cannot be fully trusted. Additionally, another participant mentioned the following:

*“You want to be accountable towards your consumer, so that they can at least find what is in the product. Because I think if you call a company and you talk to an employee and you ask, where is the soy coming from? I think the employee says: no idea”* (P310).

Next, to the call for increased transparency on, e.g., ingredients and nutritional value (see also section 3.2.2), the positioning of presenting meat alternatives (section 3.1.2) could be a potentially misleading factor. As such, retailers are considered to have some responsibility to not present meat alternatives in a confusing manner. Especially the freezer section in the supermarket appeared to be a source of confusion.

#### 3.3.3 Consumers

Participants agreed that consumers also have a responsibility (to some extent) to inform themselves, for example by reading the product label. Reading a label should inform consumers about the product characteristics and allow consumers to understand what they are buying. However, it was noted that not all consumers take the time (and thus the responsibility) to actually read the label:

*“Nowadays, everything must go fast because you have to work, and you need to heat the food quickly. I think not a lot of people, consumers, take a label and extensively read what is in this thing.”* (P310).

The level of attention given to the meat alternative label is perceived as an important factor to impact confusion. Some participants considered attention to labeling to be a subconscious process. There appeared to be consensus on the (sometimes) limited information available:

*“For me, I would like to be helped better (…) If I have to research it all myself that would not help me as a consumer, so if there is an actor in the supply chain or a brand that helps me with that, they more easily find me as a consumer”* (P437).

## 4 Discussion

The results presented in this study provide valuable insights into the labeling of meat alternatives and its perceived risk of misleading according to consumers. Three main themes arose from the focus group discussions, namely: 1. Possible misleading elements; 2. Expectation management; 3. Responsibility for meat alternatives to not be confusing and/or misleading. Consumers have various uncertainties and perceive unclarities about different aspects of meat alternatives. It seems that not only at a labeling level unclarities and uncertainties can arise, but also credence attributes (like healthiness or sustainability) of meat alternatives influence the risk of misleading and/or confusion among different consumers.

### 4.1 Misleading and confusion along the consumer journey

As the aim of this study was to investigate which aspects (and to what extent) of meat alternatives are perceived as confusing and/or misleading according to Dutch consumers, we tried to visualize where confusion or misleading can occur when consumers look at a meat alternative label. A schematic representation of the consumer journey and elements where misleading could occur is displayed in Figure 1.

**Figure 1:**
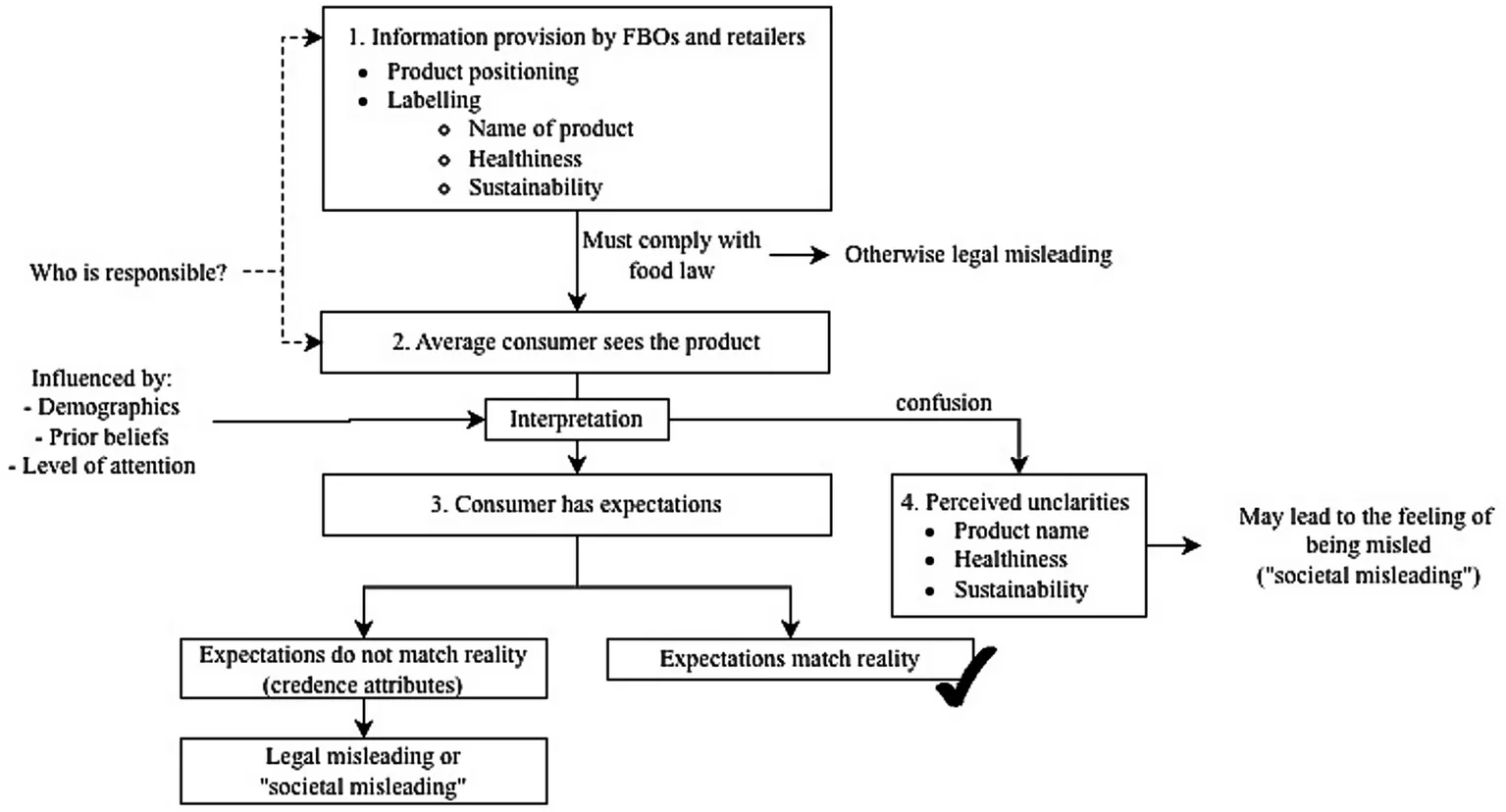
Schematic overview of the consumer journey with elements on which misleading can occur. FBOs: Food Business Operators.

When a meat alternative is put on the shelf at the retailer, it has a label containing the name of the product, nutritional information and health, nutrition and/or sustainability claims (amongst others). The information provided on the label must comply with EU food law and is overseen by national authorities. If the product does not comply with EU law, it is considered legal misleading. When a consumer enters the supermarket and sees the product, they start interpreting the information provided on the meat alternative label and draw perceptions from the context the product is placed in at the retailer. How the consumer interprets the label depends on demographics, prior beliefs and how much attention is being paid to the information available (29). During this interpretation, consumers may experience confusion when information about the product is unclear, ambiguous, or difficult to interpret. In the context of meat alternatives, such confusion may concern, for example, the identity or nature of the product, its healthiness, or its sustainability (Figure 1). Confusion is therefore conceptualized here as uncertainty arising during the interpretation of product-related information and does not necessarily imply that the information is legally misleading. However, such confusion may contribute to a subjective feeling of being misled (“societal misleading”).

The marketing and labeling of meat alternatives can straddle a fine line between conveying accurate information and creating confusion or misconceptions. As illustrated in Figure 1, confusion may arise during consumers’ interpretation of product information, for example when the product name does not clearly communicate the nature or identity of the product. This can be related to “misleadingness due to semantic confusion”, such as when meat-related terminology used for a meat alternative creates ambiguity about the product (30). Misleadingness may also arise when expectations formed during this interpretation do not correspond with consumers’ subsequent experience or with reality. For example, a sensory claim that does not correspond with the experienced sensory characteristics could constitute intra-attribute misleadingness. In addition, nutrition, health, or sustainability claims that emphasize a favorable attribute while other relevant attributes are not communicated could contribute to inter-attribute misleadingness (30). Depending on the nature and accuracy of the information, these forms of misleadingness may result in a subjective feeling of being misled (“societal misleading”) and/or constitute legal misleading.

To a certain extent, transparency from the side of the FBOs and/or retailers can aid in reducing unclarities. If the information given on unclear attributes is available on the label or accessible by a QR code for example it could reduce the feeling of being misled by consumers. Nevertheless, the amount of transparent and accurate information will probably not remove all unclarities. Demographics and prior beliefs namely create a lens through which individuals evaluate food products. These factors impact not only the initial expectations but also the overall perception of taste, quality, and satisfaction derived from consuming these products (31). Someone raised in a culture where meat is a staple might approach meat alternatives with skepticism, expecting them to fall short in terms of taste and texture. On the other hand, individuals who have ethical or health motivations to avoid animal products may approach such alternatives with more positive expectations (32). Additionally, prior exposure to advertising, social media, and word-of-mouth recommendations can shape expectations (33). If someone has heard positive reviews about a particular meat alternative; they are likely to anticipate a positive experience when trying it. On the other hand, if someone mainly heard negative experiences, people may be averse to the product, regardless of how clear and transparent the information on the label is. This was also reflected in our focus groups, where participants’ prior beliefs and expectations appeared to influence how they interpreted the information provided on meat alternative labels. Thus, our findings suggest that the potential for confusion or feeling misled does not depend solely on the information provided, but also on the consumer’s existing expectations and perceptions.

### 4.2 Marketing and credence attributes

A couple participants related their expectations of credence attributes to the green-colored packaging of many alternative products. Indeed, existing literature on this topic confirms that consumers associate the color green with “nature” and “health” (34, 35). Concerns were expressed by participants that (ingredients used in) meat alternatives would not be as sustainable and healthy as they portray them to be in their branding and marketing, for example the use of genetically modified soy. A risk of greenwashing in this product category was observed by participants. Greenwashing is the act or practice of making a product appear to be more environmentally friendly or less environmentally damaging than it really is (36). To illustrate, it was found that a green packaging color of plant-based meat alternatives were more associated with the term ‘plants’ (37). Moreover, Seo and Scammon (2017) demonstrated that incorporating environmental claims on packaging with a green color (as opposed to other colors) enhanced consumers’ perceptions of brand sustainability (38). Currently, there is no legislative framework regarding sustainability claims in the EU and an increased risk of misleading the consumer is observed. It was recommended to take the next step either through self-regulation or legal action by the European Commission (39).

As more individuals embrace plant-based diets, questions arise regarding the nutritional value and healthiness of meat alternatives. Participants in the focus groups expressed uncertainties regarding how healthy and nutrient-dense meat alternatives are but think meat alternatives are healthier than meat. This is also confirmed in a recent study, where Dutch consumers tended to view meat alternatives as being healthier compared to meat products, and incorrectly overestimated the protein content of meat alternatives (16). A survey by Van Loo, Caputo and Lusk additionally suggests that the word ‘healthy’ is highly associated with plant-based alternatives (17). Nevertheless, both sustainability and health are complex concepts that appear to be difficult to communicate to the consumer in an all-encompassing manner from a company’s perspective (40).

### 4.3 Meat-like terminology on meat alternative labels

In addition to the credence attributes, the use of meat-like terminology on labels and the positioning of meat alternatives with retailers evoked strong responses from participants. On one hand, some participants considered it definitely misleading to use meat terminology on a product that is not meat, whereas on the other hand, some participants are not bothered by it and do not consider it misleading. When comparing diet preferences and remarks on whether consumers are concerned about being misled by meat-like terminology on meat alternatives, it became evident that mainly meat eaters are concerned with buying the wrong product. People who are flexitarian, vegetarian or vegan appeared not as concerned, arguing that meat alternatives are currently very different in packaging compared to meat. Interestingly, most participants did not regard the product label as a decisive factor in grocery shopping. To contextualize these differing perceptions of meat-like terminology, the present findings can be compared with previous studies examining whether such terminology causes confusion. Although avoiding confusion and misleading consumers is central to the debate on meat alternative naming and labeling, few studies have addressed whether meat-like terminology on meat alternatives is actually confusing.

Although avoiding confusing and misleading is central to the debate on meat alternative naming and labeling, so far, few studies have addressed the question whether the meat-like terminology of meat alternatives actually is confusing. A US study reported that over 70% of participants opposed the use of terms such as “beef” for plant-based alternatives (17), whereas a large EU-wide survey by the European consumer organization (BEUC) found that only a minority of consumers (20.4%) objected to meat-like terminology when products are clearly labelled as vegetarian or vegan (18). Similarly, research in Germany indicated broad consumer support for meat-like terminology under the condition that plant-based origin is clearly indicated (78%), with only a small proportion of consumers reporting unintentional purchases of meat alternatives instead of meat (19). More recent experimental evidence adds further nuance, showing that meat-like names can increase classification errors and response times in controlled tasks, suggesting some level of cognitive ambiguity (41). At the same time, their findings indicate that consumers do not necessarily interpret such products as containing animal-derived ingredients, and that perceptions are divided regarding whether such terminology is misleading. The study also highlights that label interpretation extends beyond product names alone and is influenced by packaging, retail context, and broader product communication. An empirical study by Gleckel (2020) found that consumers are unlikely to think or believe that plant-based products that incorporating terminology referring to animal products would actually contain animal-based ingredients (15). Moreover, omitting animal-based terminology causes a decrease in the appeal of meat alternatives, resulting in consumers less willing to try meat alternatives (42).

Similar challenges have been observed for other alternative proteins, with a recent Japanese study on cell-based foods showing that different terms involve trade-offs in the information they convey. While “cell-based” was less likely to be confused with conventional foods, “cultured” and “cell-cultured” better communicated the production technology but were more susceptible to other forms of misinterpretation, highlighting that terminology that provides clarity about one product characteristic may create ambiguity about another (43).

Taken together, these studies provide context for the differing views observed among participants in the present study. The present findings similarly suggest that meat-like terminology may primarily function as a cue for consumer expectations rather than as a direct source of misunderstanding. Participants similarly indicated that they rely on the overall packaging rather than isolated terms when evaluating meat alternatives, and that meat-like names can help communicate expected taste, preparation, and use within meals. These insights are relevant in light of recent policy developments, including the 2025 European Parliament position towards restricting certain meat-related terms for plant-based products (11, 12). This development reflects ongoing efforts to improve clarity and consistency in food labeling. At the same time, the available evidence suggests that consumer interpretation is shaped by multiple elements of the label and product context. As such, the effectiveness of terminology-based restrictions versus broader labeling standards remains an important area for future consideration in the development of coherent regulatory approaches to meat alternative labeling.

### 4.4 Future outlook

Ensuring that meat alternative labeling is not misleading requires a clear legal framework supported by practical and workable policy guidelines that national authorities can consistently apply. The recent 2026 European Parliament position, which moves towards restricting the use of certain meat-related terms on plant-based products, reflects an attempt to increase clarity for consumers by limiting potentially ambiguous terminology. However, the present findings suggest that such an approach may not fully address how consumers actually interpret product labels in practice. Participants in this study emphasized that their understanding of meat alternatives is based on the overall packaging rather than on individual terms alone, indicating that isolated restrictions on terminology may have limited impact. Instead, participants pointed towards the importance of clear and visible labeling standards, particularly regarding the explicit indication that products are vegan or vegetarian and intended as meat alternatives. Consumer choice could also be supported beyond the product label itself, as the retail environment provides important contextual cues for product interpretation. Consistent and recognizable placement of meat alternatives within supermarkets, whether alongside conventional meat products or in a clearly designated section, could help consumers identify and distinguish these products. Moreover, alignment between product labeling, shelf placement and a broader retail context, could reduce ambiguity by providing consumers with consistent information at different stages of the purchasing process. This could include standardized requirements for the prominence, size, and wording of “vegan” or “vegetarian” claims on packaging, as well as consistent conventions for products designed to mimic specific meat products. Ultimately, rather than focusing solely on restricting specific terms, a more effective approach may lie in developing harmonized labeling standards alongside consistent retail communication that reduces ambiguity across both product packaging and the broader purchasing context, thereby supporting informed consumer choice.

### 4.5 Strengths and limitations

There is limited research on the misleading potential of meat alternatives that resemble meat. Findings from this study add to the existing body of literature on consumer perceptions of meat alternatives. Furthermore, they may offer several valuable in-depth insights, as well as perspectives in the political debate surrounding labeling practices of meat alternatives. Since political discussions are ongoing, this study could give grounds for larger-scale research and help in the discussion of meat-like terminology on meat alternatives within the European Union.

However, this study does not come without its limitations. First, the different aspects of meat alternatives discussed during the focus groups could differ between and within countries in the European Union and might change over time due to developments in the field of meat alternatives. Therefore, any implications and conclusions in this study are limited to the current circumstances surrounding meat alternatives in the Netherlands. In addition, the data were collected in 2022 and 2023. Since then, the market for meat alternatives has continued to evolve, with changes in product availability, product formulation, pricing, and public discourse. As a result, some perceptions and experiences reported by participants may have changed over time. Nevertheless, the transition towards more plant-based diets and the role of meat alternatives remain highly relevant topics, suggesting that the underlying insights into consumers’ perceptions continue to provide valuable contributions.

Secondly, the study sample included some notable characteristics. The focus groups were relatively small, but this allowed the participants to discuss the topics in-depth. Also, of the 25 participants in this study, 18 were female. In previous literature, women have been found to have higher preferences for meat alternatives than men (21). Furthermore, women exhibit having a greater interest in healthy eating compared to men, which could influence label and nutrition information usage (44). Therefore, the predominance of female participants may have created a biased picture of overall perceptions towards meat alternatives found in this study. In addition, the sample had a relatively high level of education, with most participants having obtained a higher education degree. A higher level of education may be associated with greater familiarity with processing and evaluating detailed product information, which could have influenced participants’ perceptions of meat alternative labeling.

## 5 Conclusion

This study investigated whether aspects of meat alternatives are perceived as confusing or misleading by consumers, with a focus on labeling practices for meat-mimicking products. While unintentional purchases of meat alternatives were not reported, many consumers still felt misled, indicating a discrepancy between the definition of misleading under EU food law and consumers’ perceptions. This study distinguishes between legal misleading and societal misleading, highlighting a potential gap in regulatory frameworks. Findings suggest that meat-like terminology enhances familiarity and appeal, providing a useful reference for sensory attributes, preparation, and usage. Policymakers need to take further steps into reconsidering labeling regulations to balance transparency with the promotion of sustainable food choices.

## Data Availability

The original contributions presented in the study are included in the article/Supplementary material, further inquiries can be directed to the corresponding author/s.
