# Supplementary material for: Seductive or deceiving? A focus group study on perspectives and expectations of consumers on meat alternative labeling practices
Source: Front Public Health. 2026 Sep 22;14:1938629. doi: 10.3389/fpubh.2026.1938629 (PMC13638586; doi:10.3389/fpubh.2026.1938629)
Supplement: Supplementary file 1 [file Table_1.DOCX]

**Demographic survey (translated from Dutch)**

Labels of meat alternatives – Focus group – Demographic survey:	Date:

Number.:

1.	What is your age?

…………………………………………………………………………………………………………………………………..

2.	What is your gender?

	Female

	Male

	Other, namely

…………………………………………………………………………………………………………………………………..

3.	What is your nationality?

…………………………………………………………………………………………………………………………………..

4.	What is your religion?

…………………………………………………………………………………………………………………………………..

	Rather not say

5.	What is your highest level of education?

	No diploma

	Primary school

	VMBO, HAVO / VWO onderbouw, MBO1

	HAVO, VWO, MBO2-4

	Bachelor (HBO/WO)

	Master (HBO/WO)

	Doctor, PhD

	Rather not say

6.	What is your yearly income?

	Less than 10.000 euro

	10.000-20.000 euro

	20.000-30.000 euro

	30.000-40.000 euro

	40.000-50.000 euro

	50.000-100.000 euro

	100.000-200.000 euro

	Over 200.000 euro

	Rather not say

7.	How often per week do you eat meat at the main meal of the day?

…………………………………………………………………………………………………………………………………..

8.	How often per week do you eat meat alternatives at the main meal of the day?

…………………………………………………………………………………………………………………………………..
