# Supplementary material for: Seductive or deceiving? A focus group study on perspectives and expectations of consumers on meat alternative labeling practices
Source: Front Public Health. 2026 Sep 22;14:1938629. doi: 10.3389/fpubh.2026.1938629 (PMC13638586; doi:10.3389/fpubh.2026.1938629)
Supplement: Supplementary file 2 [file Table_2.DOCX]

**Researcher reflexivity statement**

LK

I am a *REDACTED*, white, cisgender, straight, non-disabled female who was raised in a middle-class household in *REDACTED*. During my upbringings meat was consumed regularly during meals. At the time I did not know any better that this amount of meat consumption was normal. It was not until I started my Bachelor *REDACTED* I was confronted with the devastating effects on health and the environment of overconsuming meat. My interest grew even further during the master *REDACTED*, where more in depth knowledge was shared on the topic of meat consumption. I got the opportunity to pursue a PhD in the field of meat alternatives where I now make a living from doing research. As a result of this, I started to reduce my meat consumption when I was 19 and now, I consume meat only two to three days a week. When I eat meat, I try to find local products where the farmers take good care of their animals. On other days I replace the meat products in dishes with the corresponding meat alternative or another protein source like pulses and legumes. Consequently, I am very familiar with meat alternatives and their labels, and I am fully aware my background may influence data analysis. I will reflect on this regularly with the research team during the analysis.

RC

I am currently *REDACTED* years old and identify as a white, straight female. I grew up in *REDACTED* in the Netherlands, in a middle-class household in which meat was a central part of the diet. During this time, I have always consumed and enjoyed meat on a regular, daily basis. However, as I acquired more knowledge on the animal agriculture industry, I established strong personal feelings on animal welfare and started following a primarily plant-based diet when I was 17 years old. This diet change led to me developing a strong interest in nutrition in general. I started a bachelor Nutrition and Health at *REDACTED* in the Netherlands in 2016. Following my bachelors’ degree, I enrolled in a Masters’ program in *REDACTED* in the Netherlands in 2020, where I have been focusing on innovation of healthy, plant-based food products. During this time, my interest in meat alternatives was sparked as I consumed them frequently myself, thereby developing familiarity and experience with such products. Regarding qualitative research and focus group research specifically, my experience is limited, this research being my first personal involvement in such a study design. The methods of this study are mainly based on the variety of scientific sources mentioned in this paper, with a focus on the works of Krueger and Casey, Braun and Clarke and Creswell and Poth. I am aware that the scientific philosophical standpoint taken in this study, as well as my personal educational and background, may influence data collection and interpretation. To remain a sense of objectivity during the focus group discussions, as a moderator, I will refrain from consciously sharing or making clear my personal views in any way, which requires reflexiveness. I will reflect on the potential influence of my background and experience in a continuous process during data collection and analysis, and will discuss their potential impact throughout the research with the research team.
