# Supplementary material for: Seductive or deceiving? A focus group study on perspectives and expectations of consumers on meat alternative labeling practices
Source: Front Public Health. 2026 Sep 22;14:1938629. doi: 10.3389/fpubh.2026.1938629 (PMC13638586; doi:10.3389/fpubh.2026.1938629)
Supplement: Supplementary file 3 [file Table_4.DOCX]

**Discussion guide** (translated from Dutch to English)

Labels of Meat Substitutes – Focus Group:

Date:

Moderator:

Note-taker:

- Welcome, introduction, and moderator and note-taker introductions

- Sign consent form

- Complete demographic questionnaire

- Announcements and rules

o Audio recording of this discussion will be made

o During the transcription of this audio recording, all names will be removed, and everyone will be anonymized

o Everything said in this discussion is confidential

 You yourselves know who is in this focus group

 What is said in the discussion, and by whom, stays within the group

o There are no right or wrong opinions

 Not just answering the moderator's questions but responding to each other

 A conversation/discussion within the group

o One person speaking at a time, try to speak loudly and clearly

o You may not share the same opinion

 Respect each other's opinions

 You don't have to reach a common standpoint or decision

 Let us know if you agree/disagree with something and why, or if you have additions

 Provide your own examples when possible

o If someone speaks a lot or very little, the moderator may point it out and guide the conversation

o Discussion duration is approximately one and a half hours but may run a little over

o Turn off your mobile phone or set it to airplane mode

o Transcript/results available upon request

- Any questions?

o Turn on the recorder

o Name tags and introduction round

- Discussion Questions:

- Phase 1 – opening questions:

1. What is the first thing that comes to mind when you think of meat substitutes?

2. What do you consider a meat substitute?

 Definition, characteristics

a. Specify our definition of a meat substitute in this research: 'A product designed to replace meat during the main meal, primarily based on plant-based protein.'

3. What do you think of meat substitutes?

 Experiences

 Positive/negative aspects

a. Probe into sensory characteristics, price, health, nutritional values

b. Do you often eat meat substitutes, and why or why not?

- Phase 2 – labels & confusion/misleading:

4. What do you think of how meat substitutes are offered/promoted to consumers?

 Marketing and advertising

 In the supermarket

 Appearance

 Packaging and labeling

a. Possibly inquire: What role does the label play when you want to buy a meat substitute, what do you look for?

5. Have you ever accidentally bought a meat substitute, thinking it was real meat?

a. Inquire further: Suppose this happened to you, how would you feel about it?

6. Do you find the way meat substitutes are presented unclear/confusing or misleading? Why or why not?

 Marketing and advertising

 In the supermarket

 Appearance

 Packaging and labeling

a. Discuss the difference between confusion/uncertainty and deception

b. Do you now have a different view on whether meat substitutes are presented unclear/confusing/misleading?

- Phase 3 – examples from the supermarket:

7. What do you think of the following (labels of) meat substitutes?

a. Show different existing labels of meat substitutes

b. Possibly inquire: Of these examples, which meat substitute do you prefer and why?

c. Possibly inquire: What else would you like to see on the packaging, what would you put on it yourself? What would make it better?

8. Now that we've had this discussion, do you now view meat substitutes differently? Why or why not?

9. We would like to briefly evaluate, are there things you think would make this focus group better? Is there anything about meat substitutes that has not been discussed but you think is important?

è Stop the recorder

- Debriefing

o Words of thanks

o Emphasize anonymity and confidentiality

o As mentioned earlier, transcript/results available upon request

o Distribute cards with brief information, including contact details of the main researcher and executing researchers

**Meat alternatives discussed during phase three focus groups**

***The Vegetarian Butcher chicken pieces***

***Jumbo vegetable disc***
